# Rare Diabetic Complications: A Case of Diabetic Myonecrosis

**DOI:** 10.7759/cureus.107141

**Published:** 2026-04-16

**Authors:** Talya Jaffe, Anna Bode, Charles Pizanis

**Affiliations:** 1 School of Medicine, University of New Mexico, Albuquerque, USA; 2 Internal Medicine, University of New Mexico, Albuquerque, USA

**Keywords:** diabetes-associated focal myonecrosis, diabetic muscle infarction, endocrinology and diabetes, type 2 diabetes, uncontrolled diabetes

## Abstract

Diabetic myonecrosis, also referred to as diabetic muscle infarction, is a rare complication of uncontrolled diabetes that is characterized by spontaneous ischemia of muscle tissues, which can lead to severe pain. This condition can occur in type 1 and type 2 diabetics, most often involving the thigh or calf muscles, with the majority of literature being case reports with a few systematic reviews due to its rarity.

We present a case of a 41-year-old male with severely uncontrolled type 2 diabetes and three weeks of worsening left calf pain, elevated inflammatory markers, and myonecrosis on MRI, who was found to have probable diabetic myonecrosis and was treated with rest, pain control, and aspirin with significant clinical improvement.

As diabetic myonecrosis is a rare condition and is not often considered in the differential, there is potential for misdiagnosis or underdiagnosis of this condition. Our case report will add to the body of literature regarding this rare condition, which could increase clinician awareness of the condition, provide guidance for work-up and treatment, and contribute to future research.

## Introduction

Diabetic myonecrosis is a rare, often-overlooked complication of chronically uncontrolled diabetes mellitus. It involves painful spontaneous ischemic necrosis of skeletal muscle, typically in the thigh or calf [1]. One systematic review reported fewer than 200 cases since the first reported case in 1965 [2]. The condition occurs more commonly in females compared to males, at a mean age of 40-45 years, and occurs in both type 1 and type 2 diabetics, with some studies reporting a higher proportion of type 1 while other studies report a higher proportion of type 2 [1,2]. Of 115 patients and 166 episodes in a systematic review by Trujillo-Santos, 59% had type 1 diabetes [1]. However, in another systematic review by Horton et al., among 108 patients with diabetes, 50% had type 2 diabetes, while 41.7% had type 1 diabetes [2].

The mechanism of pathogenesis is unknown; however, several hypotheses exist, including microvascular ischemia secondary to atherosclerosis, microangiopathy, or vasculitis, leading to myocyte injury [1,2]. There is no specific standard of care; however, it has been shown that time to recovery is shorter with rest, pain control, and antiplatelet agents compared to surgical intervention [3]. Although short-term prognosis is good, diabetic myonecrosis is associated with poor long-term prognosis, including high rates of recurrence and an association with mortality within five years following diagnosis [3,4]. We report a case of a 41-year-old male patient with three weeks of refractory left lower extremity pain and swelling, who was diagnosed with and successfully treated for diabetic myonecrosis of the left posterior calf compartment. Given the rarity of this condition, it may not often be considered in the differential, increasing the potential for misdiagnosis or underdiagnosis. This case report could increase clinician awareness of diabetic myonecrosis, serve as an example for work-up and treatment, and potentially be beneficial to future research of the condition.

## Case presentation

A 41-year-old male with 24 years of uncontrolled type 2 diabetes mellitus (glycosylated hemoglobin (HbA1c) = 16.5%) complicated by peripheral neuropathy presented to the emergency department during July of 2024 with three weeks of progressive left lower extremity swelling and severe calf pain with radiation into his buttock at rest. The patient denied any trauma to the extremity, open wounds, fevers or chills, recent surgeries, or long car rides or flights. The patient reported his home regimen for his diabetes was "3 pills" for the metformin per day and 30 units nightly of long-acting insulin. However, he noted that he was not using his home medications as he felt they were ineffective. On exam, the patient was afebrile with normal oxygen saturation on room air but was intermittently tachycardic and mildly hypertensive. He was in mild distress with bilateral diminished sensation in his lower extremities, consistent with his diabetic neuropathy, as well as swelling and severe pain to palpation of the left calf, without signs of trauma or erythema. Bilateral dorsalis pedis pulses were intact with no signs of ischemia, skin discoloration, or wounds.

**Figure 1:**
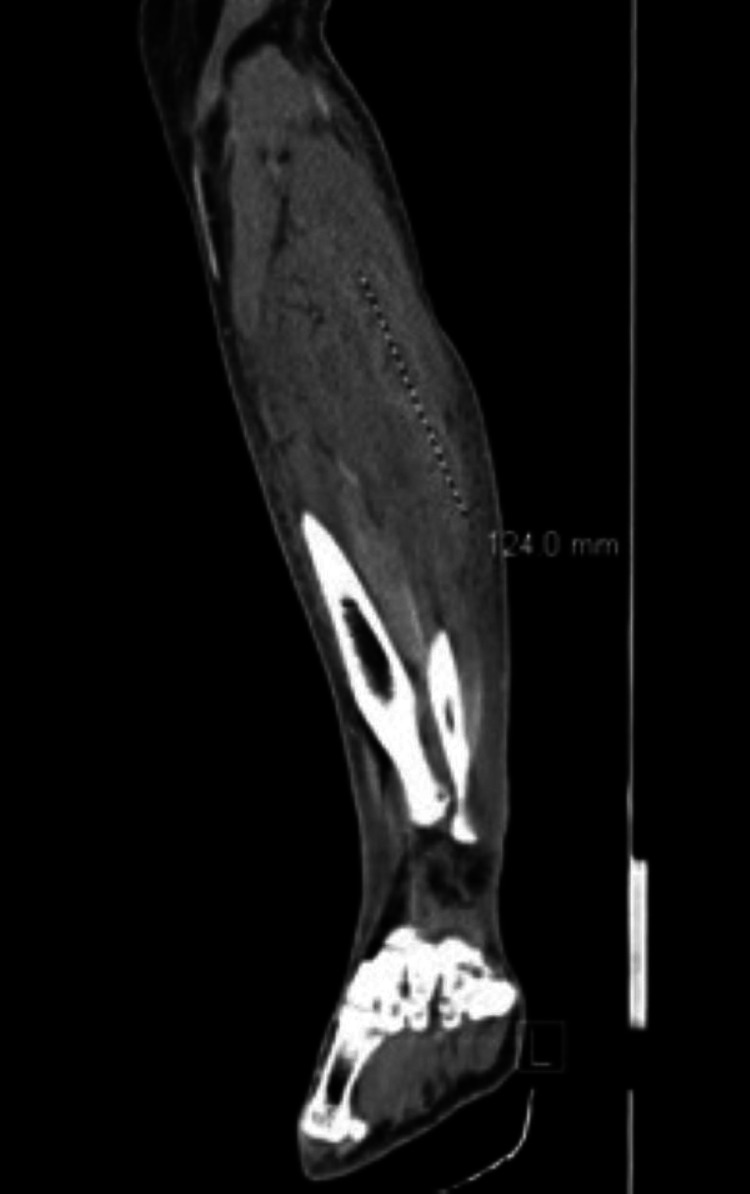
Left lower extremity CT with 12.4 cm fluid collection of the gastrocnemius visualized (depicted by dotted line), sagittal view.

**Figure 2:**
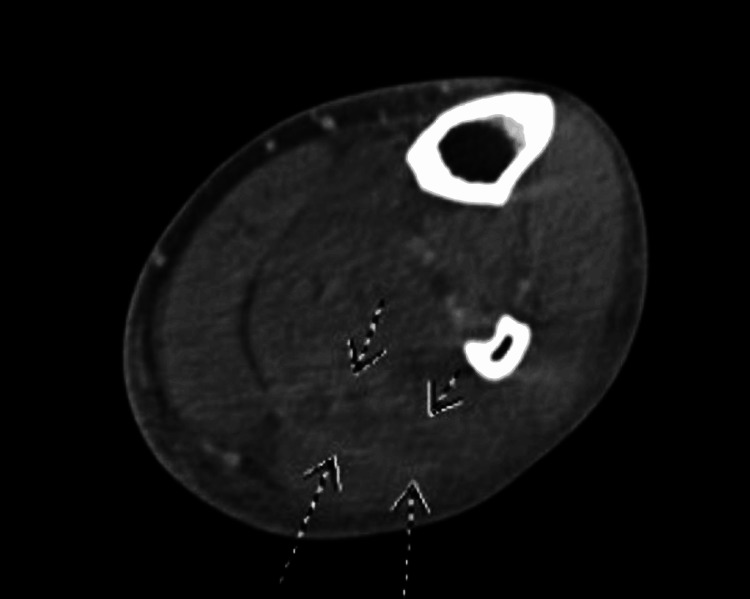
Left lower extremity CT with fluid collection visualized, transverse view.

His lab workup (Table 1) was notable for severe hyperglycemia of 643 mg/dL and elevated urinary ketones but normal pH on venous blood gas. He was treated with intravenous fluids and an insulin drip, which was transitioned to subcutaneous insulin while in the emergency room, with improvement of his hyperglycemia. Hematologic parameters were normal, but inflammatory markers were elevated with ESR at 56 mm/hr and CRP at 7.3 mg/L. Left lower extremity venous Doppler ultrasound was negative for deep vein thrombosis, while a CT of the extremity demonstrated an ill-defined, rim-enhancing, fluid collection at the lateral head of the gastrocnemius with subcutaneous edema (Figures 1, 2).

**Table 1:** Laboratory findings. WBC: white blood cells; pCO2: partial pressure of carbon dioxide; ESR: erythrocyte sedimentation rate; CRP: C-reactive protein; CK: creatine kinase; BUN: blood urea nitrogen; eGFR: estimated glomerular filtration rate.

Lab test	Result	Range
WBC	7.4	4.0 - 11.0 x 10³/uL
Lactate	1.5	0.5 - 2.2 mmol/L
Potassium (K)	3.9	3.5 - 5.0 mmol/L
Glucose	643	70 - 100 mg/dL
Sodium	128	135 - 145 mEq/L
Sodium (corrected)	137	135 - 145 mEq/L
Urine ketones	20	Negative
pH	7.45	7.35 - 7.45
pCO2	33	35 - 45 mmHg
Bicarbonate (HCO3)	17	22 - 28 mmol/L
Anion gap	19	8 - 12 mEq/L
Hemoglobin A1c	16.5	4.0 - 5.6%
ESR	56	0 - 20 mm/hr
CRP	7.3	< 3.0 mg/L
CK	134	28 - 319 unit/L
BUN	10	7 - 25 mg/dL
Creatinine	0.84	0.7 - 1.35 mg/dL
eGFR	112	mL/min/1.73 m^2^

**Figure 3:**
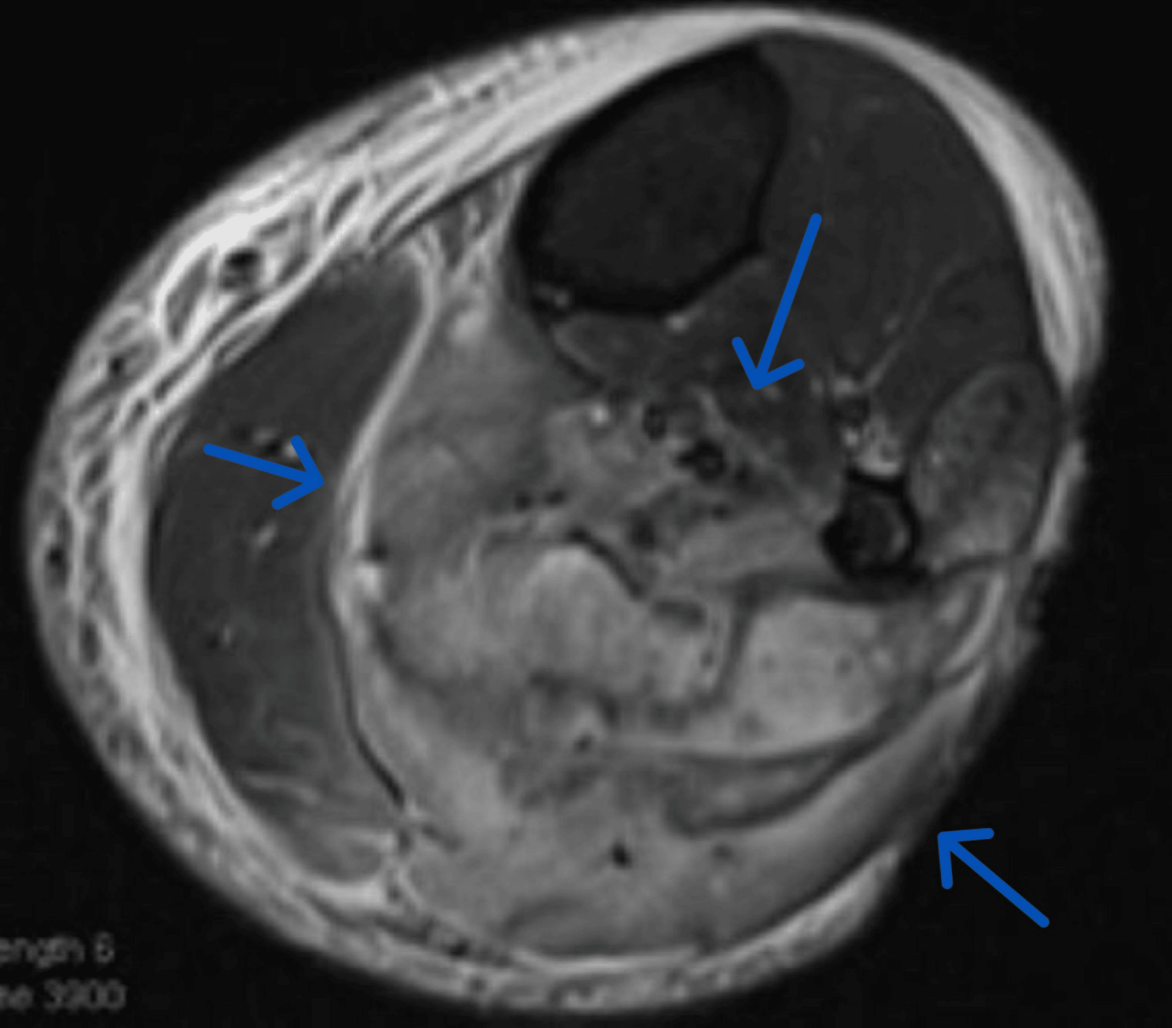
Left lower extremity MRI with abnormal posterior compartment signal.

The patient was admitted to the hospital for further workup and management. Primary differential diagnosis included infectious myositis, hematoma, and myonecrosis. The patient was assessed by general and orthopedic surgery teams, who had low concern for compartment syndrome, and it was determined that no surgical intervention was indicated. He was started on intravenous ceftriaxone 2 grams every 24 hours and vancomycin with a loading dose of 1.75 grams, followed by 1 gram every eight hours due to initial concern for infection. An MRI of the extremity was obtained, which demonstrated an abnormal signal in the posterior compartment of the left calf, most suggestive of developing myonecrosis (Figure 3). During the hospital course, he continued to be afebrile without development of erythema or leukocytosis. Infectious workup was negative, including blood cultures and methicillin-resistant Staphylococcus aureus (MRSA) nares. He received three days of ceftriaxone and two days of vancomycin total. His symptoms did not improve despite antibiotic therapy.

Given the negative infectious workup, lack of trauma, lack of systemic symptoms, and Doppler negative for deep venous thrombosis, the diagnosis of diabetic myonecrosis was made. The patient’s antibiotics were subsequently discontinued, and he was started on aspirin 81 mg daily along with a pain regimen of acetaminophen 1000 mg three times daily, methocarbamol 1500 mg three times daily, his home gabapentin 600 mg three times daily, topical lidocaine cream, and a short-term prescription of oxycodone 10 mg every six hours as needed for severe pain. Nonsteroidal anti-inflammatory drugs (NSAIDs) were considered, though avoided due to concern for risk of kidney injury with long-term or daily use in the setting of his uncontrolled diabetes. Other components of his management involved strict glycemic control, a consultation with a diabetes educator, and a continuous glucose monitor. His symptoms improved on the above regimen, and he was discharged home.

At the three-month follow-up, the patient’s HbA1c had come down to 7.8%, and he reported the pain from the myonecrosis had resolved, though he was still struggling significantly with ongoing nocturnal neuropathic pain secondary to his diabetic neuropathy. He was started on a glucagon-like peptide 1 (GLP-1) agonist, which may help improve his outcome over the next few years.

## Discussion

This case emphasizes the importance of recognizing persistent muscle pain and swelling in a patient with long-standing uncontrolled diabetes as a possible diabetic muscle infarction, or diabetic myonecrosis. This patient had a relatively typical presentation and timeline of symptoms for this condition, with the mean diabetic disease duration being around 14 years at the time of diagnosis [1]. Most cases involve the lower extremities, consistent with this patient’s gastrocnemius localization on his MRI, although the quadriceps muscles are more frequently involved than the calf muscles [4]. This is possibly due to the higher oxygen demand of a larger muscle group, increasing susceptibility to blood flow issues. Additionally, in a study by Horton et al., the majority of patients had creatine kinase in the normal range along with elevations in ESR and CRP, as did our patient [2].

The mechanism of diabetic muscle infarction is still not fully known, but it is likely due to microvascular injury with resulting inflammation. Atherosclerotic disease, diabetic microangiopathy, vasculitis with clot formation, and ischemia-reperfusion injury have all been postulated as causes contributing to muscle infarction [2]. It can also occur in the setting of chronic diabetic foot ulcers, which result in chronic inflammatory cytokine release, and is commonly seen in patients who already have other microvascular complications such as retinopathy (71%), nephropathy (57%), and/or neuropathy (55%) [5]. This patient had already been struggling with both retinopathy and neuropathy, with concern for developing nephropathy that prevented the use of NSAIDs in his case.

MRI with contrast is the imaging modality of choice for diagnosing diabetic myonecrosis, as was used in this case, but in the case of inconclusive imaging, a more invasive muscle biopsy may be performed as well. The muscle biopsy usually demonstrates loss of striation, edema, and necrotic muscle cells [4].

There are three reported modalities for treatment in diabetic myonecrosis. Conservative treatment involves rest, pain control, and improved glycemic control [2]. Medical treatment involves an attempt to address underlying microvascular pathophysiology and can include antiplatelets, NSAIDs, steroids, and even anticoagulation in cases where a patient has a hypercoagulable condition such as antiphospholipid antibody syndrome [1,2,5,6]. Low-dose aspirin (specifically 81 mg daily) is the most frequently utilized and has been shown to shorten recovery time [2]. Finally, surgical treatment, usually in the form of fasciotomy and/or tissue debridement, is a third option discussed in various cases and studies. In a review of 49 published cases of diabetic myonecrosis, medical treatment groups experienced the shortest average time to recovery compared to conservative and surgical treatment groups [3]. Average recovery lasted 5.5 weeks with medical treatment, 8.1 weeks with conservative treatment, and 13 weeks for surgical treatment. Surgical treatment should, therefore, be avoided except in refractory cases or concerns for compartment syndrome [3]. Interestingly, physical therapy may have a negative impact on healing, as studies indicate that patients who have physical therapy as part of their treatment experienced a longer average recovery time [2]. Of note, given the rarity of this condition, recommendations are mostly at the level of case reports/case series.

While most patients have good short-term outcomes with complete recovery if they receive proper medical attention, there is a high chance of recurrence, which happens in about 40% of patients and is usually contralateral [2]. The five-year mortality rate after a diabetic myonecrosis diagnosis is over 50%, and its diagnosis is often indicative of a poor long-term outcome [7]. This is likely due to overall severe underlying vascular complications due to uncontrolled diabetes, not the myonecrosis itself.

## Conclusions

Recognition of diabetic myonecrosis is paramount to initiating proper treatments and avoiding unnecessary therapies for conditions with similar presentations (e.g., antibiotics for infectious myositis). Achieving good glycemic control is the most important action to take to prevent recurrence of the condition. Additional treatment should include rest, aspirin, NSAIDs, and avoidance of surgery or aggressive physical therapy. Further study is needed to improve understanding of the mechanism of the infarction and to identify treatments that prevent recurrence and reduce the subsequent mortality risk associated with this unusual and severe diabetic complication.
